# Supplementary material for: Identification of photoperiod-induced specific miRNAs in the adrenal glands of Sunite sheep (Ovis aries)
Source: Front Vet Sci. 2022 Jul 22;9:888207. doi: 10.3389/fvets.2022.888207 (PMC9354845; doi:10.3389/fvets.2022.888207)
Supplement: Supplementary file 1 [file Data_Sheet_1.ZIP › Supplementary file/miRDeep2 file.zip!/miRDeep2 file/Know_Structure/SP42A2/oar-mir-200b.pdf]

miRBase precursor	: oar-mir-200b
Total read count	: 168
oar-miR-200b read count	: 166
remaining reads	: 2

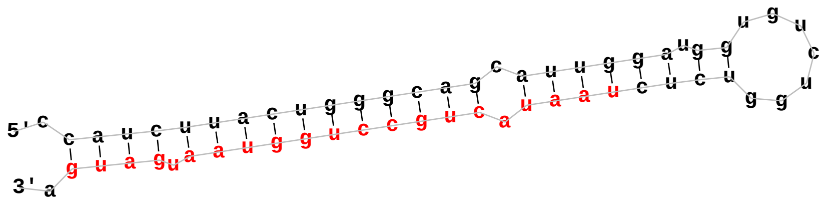

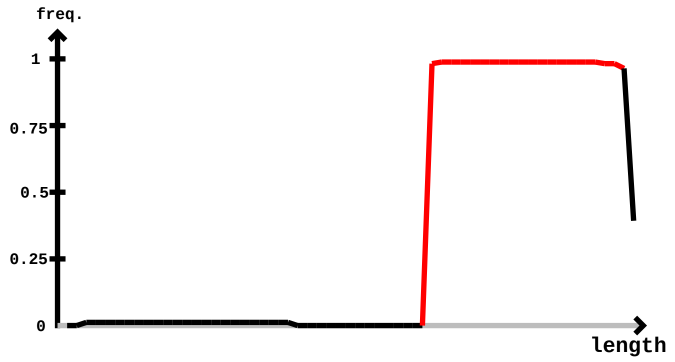


```
                                    oar-miR-200b
5'- ccaucuuacugggcagcauuggauggugucuggucucuaauacugccugguaaugaugauga   -3'    exp
    .((((((((((((((.((((((.((.......)))))))).))))))))))).)))).    reads      mm         sample
    .caucuuacugggcagcauugga...................................    2          0          seq
    ....................................uaauacugccugguaaug....    1          0          seq
    ....................................uaauacugccugguaaugau..    3          0          seq
    ....................................uaauacugccugguaaugaug.    95         0          seq
    ....................................uaauacugccugguaaugauga    66         0          seq
    .....................................aauacugccugguaaugaug.    1          0          seq
```
